# Racial Disparities in 7-Day Readmissions from an Adult Hospital Medicine Service

**DOI:** 10.1007/s40615-021-01088-3

**Published:** 2021-06-28

**Authors:** Aksharananda Rambachan, Yumiko Abe-Jones, Alicia Fernandez, Yalda Shahram

**Affiliations:** 1 Division of Hospital Medicine, University of California, San Francisco, 521 Parnassus Avenue, San Francisco, CA 94143 USA; 2 Division of General Internal Medicine, Zuckerberg San Francisco General Hospital and Trauma Center, 1001 Potrero Ave, San Francisco, 94110 CA USA

**Keywords:** Racial disparities, Readmissions, Health equity

## Abstract

**Background:**

Health systems have targeted hospital readmissions to promote health equity as there may be racial and ethnic disparities across different patient groups. However, 7-day readmissions have been understudied in adult hospital medicine.

**Design:**

This is a retrospective study. We performed multivariable logistic regression between patient race/ethnicity and 7-day readmission. Mediation analysis was performed for limited English proficiency (LEP) status. Subgroup analyses were performed for patients with initial admissions for congestive heart failure (CHF), chronic obstructive pulmonary disease (COPD), and cancer.

**Patients:**

We identified all adults discharged from the adult hospital medicine service at UCSF Medical Center between July 2016 and June 2019.

**Main Measures:**

The primary outcome was 7-day all-cause readmission back to the discharging hospital.

**Results:**

There were 18,808 patients in our dataset who were discharged between July 2016 and June 2019. A total of 1,297 (6.9%) patients were readmitted within 7 days. Following multivariable regression, patients who identified as Black (OR 1.35, 95% CI 1.15–1.58, p <0.001) and patients who identified as Asian (OR 1.26, 95% CI 1.06–1.50, p = 0.008) had higher odds of readmission compared to white patients. Multivariable regression at the subgroup level for CHF, COPD, and cancer readmissions did not demonstrate significant differences between the racial and ethnic groups.

**Conclusions:**

Black patients and Asian patients experienced higher rates of 7-day readmission than patients who identified as white, confirmed on adjusted analysis.

## Introduction

Health equity will only be achieved when healthcare outcomes do not vary based on patient social characteristics, such as gender, race, ethnicity, geographic location, and socioeconomic status. Readmission is a significant outcome for patients and health systems, and 30-day readmission has been associated with racial disparities [1–3]. Previous studies have demonstrated that among Medicare beneficiaries, Black patients were more likely to be readmitted than white patients, in general, and specifically after hospitalization for acute myocardial infarction, pneumonia, and congestive heart failure [1, 2]. Using a socioecological approach, we recognize that disparities in readmission may be due to individual, interpersonal, organizational, community, and public policy-level factors [4]. Implicit bias, individual racism, and structural racism may all contribute to disparities in readmission rates.

Over the past decade, readmission reduction has become a national policy focus with associated penalties for hospitals. For the individual patient, a readmission may reflect poor communication from their medical team and has profound implications on the individual’s health, with some evidence of higher associated in-hospital mortality after readmission [5]. However, less is known about disparities in 7-day readmissions. Previous studies have demonstrated that 7-day readmissions reflect a similar patient mix to 30-day readmissions but that 7-day readmissions may be a better measure of hospital care [6–10]. Readmissions after 7 days may be driven by factors beyond a hospital’s control. In the realm of health equity, studying 7-day, as opposed to 30-day readmissions, may better capture modifiable factors, diagnostic error, clinical decision making, and implicit bias from the provider. We examined 7-day readmissions for adults discharged from our hospital medicine service, with an analysis focused on differences between racial and ethnic groups.

## Methods

### Study Population and Data Collection

This study was performed at the University of California, San Francisco (UCSF) medical center, a 600-bed diverse, urban academic teaching hospital. All data was collected from Clarity, derived from our electronic health record, Epic. We identified all adults ≥18 years old discharged from the hospital medicine service between July 2016 and June 2019. Given the analysis of readmission, we excluded patients who died during hospitalization, were under observation status, or were transferred to a psychiatric facility.

### Primary Outcome

The primary outcome was 7-day all-cause readmission back to the discharging hospital. All-cause readmission was selected to be consistent with institutional quality metrics and national guidance from the Centers for Medicare and Medicaid Services [1]. 7-day readmission was utilized as opposed to 30-day readmission because it is more amendable hospital-based interventions prior to discharge [7, 9].

### Primary Predictor

The primary predictor was self-reported race/ethnicity, categorized as white, Black, LatinX, Asian, Native Hawaiian or Pacific Islander, American Indian or Alaska Native, or other/unknown as reported to the hospital registrar upon patient registration. Other/unknown included patients listed as other or who were otherwise unspecified. Patients were classified as LatinX if they had Hispanic as their documented ethnicity. These were all self-identified, combined racial/ethnic categories. These groupings are based on US Census Bureau definitions and were chosen to be consistent with the existing literature [11, 12]. All of these racial classifications represent socially defined groupings and serve as a surrogate for racism and structural determinants of health. They are not meant to reflect any genetic differences between these patient groups [13]. The primary outcome was a 7-day readmission back to the discharging hospital.

### Covariate Data Selection

Additional data were collected to assess for factors associated with a 7-day readmission adjusted for patient demographics including age, gender, limited English proficiency (LEP) status, housing status, and payor status. Patients were classified as LEP if they required an interpreter and had a language other than English listed as their primary language. If a patient did not meet these two specific criteria, they were classified as non-LEP. Payor or insurance status was broken down into four categories: Medicare, MediCal (California’s Medicaid), private insurance, or other (including uninsured). Hospitalization and health system factors included length of stay, intensive care unit management, teaching vs. hospitalist service, discharge disposition, and medical comorbidity index. Discharge dispositions included home, home with support, skilled nursing facility/subacute rehabilitation, acute rehabilitation, assisted living, left against medical advice, hospice, or jail/prison. Our medical comorbidity index was the Agency for Healthcare Research and Quality (AHRQ) Elixhauser readmission index. This index utilizes 30 comorbidity variables based on ICD-10 codes to assess the risk of readmission [14].

### Statistical Analysis

All analysis was performed using Stata software version 16 (StataCorp LLC, College Station, Texas). Patient demographic, hospitalization, and health system factors were stratified by race and LEP status. Within-group comparisons (Table 1) were performed using ANOVA testing. For regression analyses, we fit a multivariable logistic regression model for the association between race/ethnicity and 7-day readmission. The covariates included in the model were self-identified race/ethnicity, LEP status, housing status, payor status, age, gender, length of stay, whether they were admitted to the ICU and/or a teaching service, AHRQ Elixhauser comorbidity index, and disposition on discharge. These were selected for our model because they were a priori associated with 7-day readmission [15]. Subgroup analyses were performed on three subsets: patients with initial admissions for congestive heart failure (CHF), chronic obstructive pulmonary disease (COPD), and cancer. Cancer refers to an admission with a primary ICD-10 for any cancer-related diagnosis or treatment for cancer. For the subgroup analysis, given the reduced sample size, the other/unknown category included Native Hawaiian or Pacific Islander, American Indian or Alaska Native patients, other, or unspecified. We examined the interactions between LEP status and race. We also performed a mediation analysis based on Baron & Kenny’s four-step model to calculate the proportion of the association between race and readmissions explained by the potential mediator, limited English proficiency [16].

**Table 1:** Baseline patient characteristics discharged from the hospital medicine service by race/ethnicity^a^

Covariate *n*, (%)	Total n = 18,808	white n = 8,503	Asian n = 3,724	Black n = 2,937	LatinX n = 2,195	NH/PI^b^ n = 223	AI/AN^c^ n = 128	Other n = 1,098
Age, mean (SD)	59.8 (19.3)	59.2 (18.7)	69.0 (18.5)	55.2 (16.7)	52.3 (20.0)	67.8 (18.7)	53.6 (17.3)	58.3 (20.3)
Male gender	9,639 (51.3)	4,498 (52.9)	1,746 (46.9)	1,458 (49.6)	1,173 (53.4)	117 (52.4)	69 (53.9)	578 (52.6)
LEP^d^ status	2,893 (15.4)	303 (3.6)	1,761 (47.3)	13 (0.4)	578 (26.3)	53 (23.8)	1 (0.8)	184 (16.8)
Teaching service	13,115 (69.7)	5,947 (69.9)	2,584 (69.4)	2,039 (69.4)	1,564 (71.3)	153 (68.6)	84 (65.6)	748 (68.1)
Housing insecure	2,481 (13.2)	1,303 (15.3)	111 (2.9)	715 (24.3)	221 (10.1)	7 (3.1)	27 (21.1)	97 (8.8)
ICU admission	2,702 (14.4)	1,278 (15.0)	504 (13.5)	411 (14.0)	287 (13.1)	37 (16.6)	24 (18.8)	161 (14.7)
Comorbidity index, mean (SD)	25.1 (17.5)	23.8 (17.0)	26.2 (17.3)	28.3 (18.8)	23.9 (16.9)	25.3 (17.5)	30.6 (21.1)	24.4 (17.4)
LOS^e^ mean (SD)	6.7 (9.8)	6.7 (8.9)	6.0 (8.7)	7.3 (11.6)	6.9 (9.0)	6.8 (15.7)	9.3 (12.6)	7.2 (12.3)
Insurance status								
Medicare	9,307 (49.5)	4,193 (49.3)	2,429 (65.2)	1,321 (45.0)	732 (33.4)	129 (57.9)	44 (34.4)	459 (41.8)
MediCal	5,337 (28.4)	2,008 (23.6)	607 (16.3)	1,328 (45.2)	949 (43.2)	60 (26.9)	47 (36.7)	338 (30.8)
Private	3,893 (20.7)	2,181 (25.7)	664 (17.8)	249 (8.5)	470 (21.4)	33 (14.8)	34 (26.6)	262 (23.9)
Other	271 (1.4)	121 (1.4)	24 (0.6)	39 (1.3)	44 (2.0)	1 (0.5)	3 (2.3)	39 (3.6)

^a^within-group comparisons were all significant except for ICU and teaching service

^b^NH/PH, Native Hawaiian or Pacific Islander

^c^AI/AN, American Indian or Alaska Native

^d^LEP, limited English proficiency

^e^LOS, length of stay

## Results

There were 18,808 patients in our dataset who were discharged between July 2016 and June 2019. Representative of our region’s diversity, white patients comprised the largest group at 45%, with Asian 20%, Black 16%, LatinX 12%, Native Hawaiian or Other Pacific Islander 1%, American Indian or Alaska Native 1%, and other/unknown 6%. Between the different racial groups, there were significant baseline differences in demographic and clinical characteristics. Patients who identified as Asian were the oldest group with a mean age of 69 years and patients who identified as LatinX were the youngest at 52 years, with the mean overall age of 59 years. Patients who identified as Asian had the highest proportion of LEP at 47%, compared to 26% for patients who identified as LatinX, 4% for patients who identified as white, and <1% for patients who identified as Black. American Indian or Alaska Native patients had the highest mean comorbidity index and longest mean length of stay. Finally, there were significant differences in insurance, with patients who identified as Black or LatinX having the highest proportion of enrollees in MediCal, with patients who identified as Asian having the most enrollees in Medicare (Table 1).

A total of 1,297 (6.9%) patients were readmitted within 7 days. Patients who identified as white had a 6.4% 7-day readmission rate. Patients who identified as Native Hawaiian or Other Pacific Islander (2.7%) or other/unknown (5.2%) had lower 7-day readmission rates. Patients who identified as LatinX (6.6%), Asian (7.2%), Black (8.9%), or American Indian or Alaska Native (10.9%) had higher readmission rates. For the subgroups of COPD, CHF, and cancer readmissions, patients who identified as Black had the highest rates compared to all other racial/ethnic groups (Table 2).

**Table 2:** Unadjusted 7-day readmission results by diagnoses

Diagnosis	Overall *n*, (%)	White	Asian	Black	LatinX	Other^a^	p value	LEP^b^	Non-LEP^*^	p value
All diagnoses	1,297 (6.9)	545 (6.4)	268 (7.2)	262 (8.9)	145 (6.6)	77 (5.3)	< 0.001	174 (6.0)	1,123 (7.1)	0.042
CHF	25 (6.6)	9 (6.1)	2 (2.1)	12 (13.6)	0 (0.0)	2 (7.7)	0.018	1 (1.2)	24 (8.2)	0.022
COPD	46 (7.8)	17 (7.1)	2 (2.1)	22 (12.9)	3 (6.4)	2 (5.7)	0.026	2 (2.4)	44 (8.7)	0.046
Cancer	146 (8.3)	58 (7.8)	41 (10.5)	22 (11.9)	18 (6.0)	7 (5.1)	0.045	30 (8.6)	116 (8.2)	0.823

^a^For this table, other included Native Hawaiian or Other Pacific Islander, Alaska Native or American Indian, other, and unknown

^b^LEP, limited English proficiency

Following multivariable logistic regression, we found a statistically significant association between race/ethnicity and 7-day readmission (Table 3). Controlling for covariates, the odds ratio (OR) for a 7-day readmission for patients who identified as Black was 1.35 (95% CI 1.15–1.58, p <0.001) and for patients who identified as Asian was 1.26 (95% CI 1.06–1.50, p = 0.008). The readmission odds for other racial/ethnic groups did not differ significantly from patients who identified as white. While the bivariate analyses were significant, multivariable regressions at the subgroup level for CHF, COPD, and cancer readmissions did not demonstrate significant differences between the groups.

**Table 3:** Multivariable logistic regression between race/ethnicity and a 7-day readmission

Covariate	Adjusted OR for a 7-day readmission (95% CI)	p value
Race/ethnicity		
White	reference	-
Black	1.35 (1.15–1.58)	< 0.001
Asian	1.26 (1.06–1.50)	0.008
LatinX	1.11 (0.91–1.36)	0.284
Alaska Native or American Indian	1.71 (0.97–3.01)	0.066
Native Hawaiian or Other Pacific Islander	0.44 (0.19–1.00)	0.051
Other/unknown	0.83 (0.62–1.09)	0.190
Language		
Non-LEP^a^	Reference	-
LEP	0.88 (0.80–0.97)	0.012

^a^LEP, limited English proficiency

Patients with limited English proficiency had slightly reduced odds of readmission with an OR of 0.88 (95% CI 0.80–0.97, p = 0.012). The interaction between race and limited English proficiency was nonsignificant for Black and Asian patients and the mediation analysis assessing the effect of the potential mediator, limited English proficiency status, also did not demonstrate statistical significance. The interaction between race and insurance status was also not significant.

## Discussion

We found that patients who identified as Black and patients who identified as Asian were significantly more likely to be readmitted within 7 days after discharge from our hospital medicine service compared to patients who identified as white, controlling for demographic, clinical, and hospitalization-based variables. While previous studies have examined racial disparities in 30-day readmissions overall and 7-day readmissions among a pediatric population, our analysis of 7-day readmissions is novel for the field of adult hospital medicine [17].

A readmission reflects a multifactorial process, driven by many factors outside of a hospital’s control. Previous studies have demonstrated racial differences in 30-day readmissions. Using Medicare data, researchers have found higher odds of readmission for patients who identified as Black across various medical diagnoses [18, 19]. Our findings were consistent with this. Prior research has indicated that site of care is an important determinant of readmission disparities, demonstrating the importance of evaluating systems. Large urban teaching hospitals, like our study site, which serve a higher proportion of patients who identify as Black, were associated with higher odds of readmission for these patients [19]. The findings of this study confirm that racial disparities persist on a shorter timeline, 7 days, after discharge. The implications of this finding are relevant to hospitals trying to minimize financial penalties associated with readmission and give light to the importance of promoting greater health equity for minority patient populations.

There is less evidence regarding disparities in readmissions for patients who identify as Asian. Previous studies have demonstrated lower rates of 30-day readmission for patients who identify as Asian for specific diagnoses including diabetes [18]. The patients who identified as Asian in our analysis were older, more likely to be on Medicare, and had higher rates of readmission for cancer but not COPD or CHF. They also had a higher mean comorbidity index, which was adjusted for in our model. It is possible that the comorbidity index did not fully account for their underlying illnesses, making them more predisposed to readmission.

While a higher readmission rate has been documented previously for LatinX patients in the literature, we did not find a significantly different rate for the LatinX patients in our study [18]. The youngest patients in our analysis identified as LatinX. It is unclear if age protected this specific population from higher readmissions. We found that Alaska Native or American Indian patients had among the highest readmission rate in both the unadjusted results and regression analysis, but this was not statistically significant. It is likely that this specific analysis was underpowered with only 128 patients. Prior analyses of differences in 30-day readmissions for Native Hawaiian and Pacific Islander subgroups have demonstrated varied results, and we did not find significant differences for this group in our analysis [20].

We also evaluated the relationship between race and limited English proficiency status to assess the potential role that language played in readmission odds. Prior studies have demonstrated varied associations between LEP status and readmission [21, 22]. However, for our study, both the interaction between race and language, as well as the mediation analysis, yielded nonsignificant results. Within the context of this study, we interpret this to mean that, irrespective of language, a patient’s self-identified race/ethnicity was independently associated with readmission.

Studying 7-day readmission after discharge may be preferable to a 30-day readmission. The greatest hospital variation in readmissions among Medicare patients occurred between 1 and 7 days post discharge [10]. The corollary is that readmissions that occur after 7 days, including the more standard 30-day readmission, reflect a broader community and social factors out of the control of the discharging team and hospital [23]. Analyzing readmissions on a shorter timeline allows for more robust hospital-based interventions [6, 7]. On the part of the clinician, 7-day readmissions may better capture modifiable factors. This becomes particularly relevant in the context of identifying and reducing racial and ethnic disparities.

Our study has several limitations including being a single-center study, limited subgroup sample size, and missing information regarding readmission to other hospitals, primary care follow-up, hospitalist communication, and income data. Many studies of readmissions are limited by patients who are readmitted to different hospitals. This is common in the San Francisco Bay Area, with multiple medical facilities within a few miles of each other. We were unable to include information regarding primary care follow-up after hospitalization. Although some data were available, due to many patients receiving primary care outside of the UCSF system, no reliable analysis was performed. The impact of having reliable follow up with primary care physicians is well documented and would help to add to the understanding about the disparities measured in this study. Additionally, we recognize the importance of hospitalist communication with the patient, specifically the behavior clinicians exhibit during the hospitalization and at the time of discharge. We were unable to capture this variable in our analysis. However, implicit bias, racism, and poor communication exist in healthcare providers and would certainly impact the safety of a given discharge and the probability of readmission. Finally, we did not have access to income data. Insurance status was included in our study, and Medicaid may serve as a rough proxy for lower socioeconomic status. However, the intersection of race, income, and insurance status is well documented and would be an important focus in future studies [24, 25].

## Conclusion

Patients who identify as Black and patients who identify as Asian experienced higher rates of a 7-day readmission than patients who identified as white confirmed on adjusted analysis. Racial disparities in 7-day readmissions from adult hospital medicine services have not been previously characterized, but these findings align with known racial disparities in 30-day readmissions. We were unable to specifically study experiences of racism and discrimination experienced by patients. We emphasize that race, as defined in this study, is a proxy for racism and structural determinants. Because 7-day readmissions may be more modifiable and impacted by factors like clinician bias, these results require further investigation into causal pathways.
